# Supplementary material for: A Likelihood Approach for Real-Time Calibration of Stochastic Compartmental Epidemic Models
Source: PLoS Comput Biol. 2017 Jan 17;13(1):e1005257. doi: 10.1371/journal.pcbi.1005257 (PMC5240920; doi:10.1371/journal.pcbi.1005257)
Supplement: S1 File — (TAR.GZ) [file pcbi.1005257.s014.tar.gz › HSPH_Online-SI-Revision/IPoi09/n10-mild/n10-mild_BENCH_table8.pdf]

1	R0	{1.25046, 6.80278}	{1.7612, 13.0663}	{{1.68954, 11.6019}, {1.79436, 13.7237}}	{40.8446, 92.0724}	inf	102.	495.627	{190., 1003.}	385.909	0.615466	newly	103.	244.271	{100., 470.}	141.896	0.329254	Reff	0.913086	0.671232	{0.534899, 1.01305}	34.011	ar	3516.	7156.75	{6717., 7383.}	103.548
2	R0	{1.32013, 4.45847}	{2.12117, 12.1677}	{{2.12117, 12.1677}, {2.12117, 12.1677}}	{60.6789, 172.912}	inf	44.	288.615	{116., 640.}	555.944	0.724678	newly	57.	141.288	{58., 328.}	151.721	0.316992	Reff	0.755774	0.407775	{0.365053, 0.457748}	46.0455	ar	4420.	8275.15	{8177., 8375.}	87.2207
3	R0	{1.31785, 5.36796}	{2.28288, 11.2606}	{{2.28112, 11.2541}, {2.28112, 11.2541}}	{73.2274, 109.773}	inf	84.	237.	{66., 397.}	190.829	0.318817	newly	97.	124.852	{34., 211.}	84.9179	0.279857	Reff	0.69253	0.354662	{0.318444, 0.384141}	48.7875	ar	4942.	8586.78	{8503., 8681.}	73.7511
4	R0	{1.38801, 6.16031}	{2.2277, 15.0861}	{{2.2277, 15.0861}, {2.2277, 15.0861}}	{60.496, 144.892}	inf	76.	531.228	{201., 922.}	598.984	0.775884	newly	81.	220.596	{85., 400.}	174.464	0.368993	Reff	0.752718	0.432588	{0.344625, 0.480292}	47.0358	ar	4790.	8494.67	{8408., 8602.}	77.3417
5	R0	{1.27955, 11.57}	{1.6411, 19.8325}	{{1.6411, 19.8325}, {1.6411, 19.8325}}	{28.256, 71.4131}	inf	144.	454.432	{161., 823.}	216.704	0.441392	newly	92.	154.486	{51., 282.}	83.725	0.248604	Reff	0.976553	0.696132	{0.581278, 0.869455}	29.43	ar	3460.	6654.59	{6451., 6871.}	92.3293
6	R0	{1.22184, 4.34395}	{1.82588, 7.1185}	{{1.78724, 6.63253}, {1.83586, 7.22097}}	{49.4366, 63.8716}	inf	60.	56.6939	{11., 117.}	61.1565	0.305982	newly	80.	43.4694	{8., 104.}	63.8776	0.469684	Reff	0.820343	0.47843	{0.448317, 0.510436}	41.6793	ar	3524.	7432.18	{7210., 7587.}	110.902
7	R0	{1.23856, 4.59068}	{1.88059, 9.64917}	{{1.88096, 9.64595}, {1.88096, 9.64595}}	{51.8369, 110.191}	inf	59.	165.614	{61., 305.}	183.475	0.343426	newly	87.	100.659	{34., 174.}	66.3271	0.254772	Reff	0.795403	0.472207	{0.432809, 0.519333}	40.633	ar	3855.	7636.5	{7468., 7775.}	98.0934
8	R0	{1.34531, 10.6689}	{1.99924, 16.1869}	{{1.97176, 15.6958}, {2.05052, 16.7932}}	{48.6079, 51.7208}	inf	248.	581.41	{257., 1130.}	137.229	0.326143	newly	164.	228.689	{92., 443.}	60.7757	0.201829	Reff	0.83611	0.512198	{0.436556, 0.648759}	38.7403	ar	4852.	7963.2	{7820., 8089.}	64.1219
9	R0	{1.31775, 5.19226}	{2.30108, 15.555}	{{2.30108, 15.555}, {2.30108, 15.555}}	{74.6219, 199.581}	inf	73.	588.709	{267., 1101.}	706.451	0.853743	newly	85.	234.491	{112., 423.}	177.882	0.390172	Reff	0.713693	0.419403	{0.335037, 0.484377}	46.8067	ar	4708.	8626.	{8540., 8709.}	83.2201
10	R0	{1.25916, 6.89207}	{1.76659, 10.896}	{{1.76659, 10.896}, {1.76659, 10.896}}	{40.2991, 58.0947}	inf	69.	195.421	{74., 405.}	187.033	0.369348	newly	65.	109.5	{43., 226.}	87.9757	0.225796	Reff	0.845904	0.534923	{0.482102, 0.586861}	36.9814	ar	3823.	7210.05	{7006., 7377.}	88.5967
11	R0	{1.20377, 5.46566}	{1.68297, 7.5059}	{{1.68297, 7.5059}, {1.68297, 7.5059}}	{39.8083, 37.3283}	inf	56.	70.2632	{31., 126.}	52.7256	0.196633	newly	56.	53.2368	{23., 97.}	42.6222	0.209852	Reff	0.847334	0.540109	{0.512464, 0.5712}	36.2578	ar	3114.	6873.16	{6683., 7017.}	120.718
12	R0	{1.27878, 2.55804}	{2.04169, 8.65633}	{{2.03307, 8.59564}, {2.06386, 8.81239}}	{59.6593, 238.397}	inf	4.	100.717	{26., 181.}	2417.92	1.23254	newly	9.	66.1887	{16., 115.}	635.849	0.689002	Reff	0.778393	0.404982	{0.375305, 0.433654}	47.9721	ar	3930.	8090.62	{7980., 8201.}	105.868
13	R0	{1.41227, 5.45696}	{2.29532, 19.5049}	{{2.23915, 18.4504}, {2.30371, 19.6625}}	{62.5268, 257.432}	inf	53.	887.692	{392., 1652.}	1574.89	1.16425	newly	51.	296.8	{136., 577.}	481.961	0.696866	Reff	0.744831	0.508069	{0.365368, 0.760915}	42.2688	ar	4822.	8608.03	{8496., 8703.}	78.5158
14	R0	{1.32917, 7.27593}	{2.22947, 17.5547}	{{2.21213, 17.2871}, {2.23964, 17.6755}}	{67.7339, 141.271}	inf	144.	571.231	{221., 1094.}	296.688	0.527713	newly	106.	208.477	{71., 392.}	108.287	0.272648	Reff	0.755633	0.448454	{0.356999, 0.52542}	48.1377	ar	4758.	8514.77	{8409., 8608.}	78.9569
15	R0	{1.31629, 9.97481}	{1.78225, 11.7348}	{{1.78225, 11.7348}, {1.78225, 11.7348}}	{35.3995, 17.6443}	inf	282.	272.3	{111., 505.}	46.0284	0.216523	newly	192.	140.175	{60., 254.}	43.6328	0.260224	Reff	0.872437	0.538712	{0.488337, 0.605609}	38.2521	ar	4543.	7276.7	{7055., 7396.}	60.1739
16	R0	{1.36735, 5.74166}	{2.17091, 14.1414}	{{2.1255, 13.6009}, {2.22641, 14.8348}}	{58.7679, 146.295}	inf	112.	467.381	{206., 746.}	317.304	0.55671	newly	104.	202.857	{85., 333.}	103.755	0.268572	Reff	0.782534	0.415917	{0.361051, 0.462296}	46.85	ar	4516.	8394.49	{8254., 8533.}	85.8833
17	R0	{1.27524, 13.2396}	{1.5294, 12.0632}	{{1.44476, 10.1243}, {1.5767, 12.7726}}	{19.9302, 8.88538}	inf	176.	562.737	{371., 725.}	219.737	0.488622	newly	112.	307.895	{189., 404.}	174.906	0.425641	Reff	1.09887	0.907871	{0.737896, 1.15205}	20.4458	ar	3731.	6040.84	{5431., 6332.}	61.9095
18	R0	{1.21884, 7.71545}	{1.68876, 11.4699}	{{1.6568, 10.5289}, {1.68954, 11.6019}}	{38.5547, 48.6609}	inf	93.	373.923	{140., 736.}	302.399	0.521297	newly	79.	207.019	{72., 458.}	166.431	0.352069	Reff	0.870374	0.653368	{0.549269, 0.821285}	27.9195	ar	3080.	6816.17	{6641., 7056.}	121.304
19	R0	{1.34879, 6.71464}	{2.1911, 12.1462}	{{2.18735, 12.1087}, {2.18735, 12.1087}}	{62.4492, 80.8912}	inf	64.	142.811	{57., 240.}	128.626	0.292566	newly	49.	68.3774	{25., 118.}	68.2711	0.219153	Reff	0.757615	0.36324	{0.336633, 0.388692}	52.0549	ar	4585.	8424.25	{8356., 8507.}	83.7349
20	R0	{1.37284, 5.75036}	{2.21449, 13.2892}	{{2.21449, 13.2892}, {2.21449, 13.2892}}	{61.3072, 131.102}	inf	131.	430.439	{157., 779.}	228.579	0.428057	newly	141.	196.526	{70., 346.}	69.516	0.225239	Reff	0.7784	0.402144	{0.341031, 0.478551}	48.5138	ar	4750.	8470.14	{8335., 8572.}	78.3187
21	R0	{1.29938, 5.36419}	{2.12117, 12.1677}	{{2.12117, 12.1677}, {2.12117, 12.1677}}	{63.2448, 126.832}	inf	52.	288.615	{116., 640.}	455.03	0.652128	newly	62.	141.288	{58., 328.}	133.716	0.290807	Reff	0.755719	0.407775	{0.365053, 0.457748}	46.0415	ar	4337.	8275.15	{8177., 8375.}	90.8036
22	R0	{1.31882, 7.47626}	{1.94077, 13.9712}	{{1.94617, 14.0718}, {1.94617, 14.0718}}	{47.1595, 86.8748}	inf	105.	424.489	{206., 786.}	304.397	0.545254	newly	86.	190.319	{90., 341.}	125.161	0.300903	Reff	0.86897	0.498756	{0.43964, 0.578596}	42.6038	ar	3977.	7823.38	{7709., 7934.}	96.7157
23	R0	{1.26215, 3.46375}	{1.95675, 9.79954}	{{1.95791, 9.82151}, {1.95791, 9.82151}}	{55.0327, 182.917}	inf	13.	187.356	{57., 230.}	1341.2	0.988818	newly	14.	114.667	{30., 134.}	719.048	0.720852	Reff	0.807776	0.455244	{0.4047, 0.477143}	44.5658	ar	3607.	7862.2	{7734., 7999.}	117.971
24	R0	{1.16712, 4.19464}	{1.67732, 9.44073}	{{1.67732, 9.44073}, {1.67732, 9.44073}}	{43.7144, 125.067}	inf	40.	99.9737	{35., 202.}	156.645	0.33455	newly	56.	64.2632	{21., 117.}	55.2632	0.216039	Reff	0.834491	0.55864	{0.516279, 0.605009}	33.0562	ar	3000.	6805.08	{6638., 6961.}	126.836
25	R0	{1.22751, 3.34517}	{1.7356, 7.10024}	{{1.7356, 7.10024}, {1.7356, 7.10024}}	{41.3919, 112.253}	inf	37.	140.128	{46., 348.}	280.527	0.48241	newly	58.	109.103	{33., 286.}	105.703	0.264517	Reff	0.861344	0.535166	{0.49239, 0.609543}	37.8685	ar	3086.	7077.31	{6931., 7297.}	129.336
26	R0	{1.18747, 5.88083}	{1.76659, 10.896}	{{1.76659, 10.896}, {1.76659, 10.896}}	{48.7692, 85.28}	inf	33.	113.947	{41., 209.}	248.804	0.425083	newly	33.	64.3947	{23., 108.}	112.998	0.257459	Reff	0.772805	0.515212	{0.476096, 0.562306}	33.3322	ar	3614.	7210.05	{7006., 7377.}	99.5034
27	R0	{1.29023, 4.76202}	{2.12134, 14.6763}	{{2.12134, 14.6763}, {2.12134, 14.6763}}	{64.4156, 208.195}	inf	18.	462.868	{189., 792.}	2471.49	1.33141	newly	24.	197.623	{87., 355.}	723.428	0.834152	Reff	0.74614	0.43766	{0.385447, 0.518455}	41.3624	ar	4245.	8283.74	{8194., 8375.}	95.141
28	R0	{1.2258, 12.9056}	{1.6411, 19.8325}	{{1.6411, 19.8325}, {1.6411, 19.8325}}	{33.8799, 53.6736}	inf	123.	276.676	{78., 604.}	136.19	0.318261	newly	72.	92.7568	{26., 196.}	66.1411	0.248265	Reff	0.891402	0.621006	{0.554364, 0.723561}	30.3338	ar	3639.	6654.59	{6451., 6871.}	82.8688
29	R0	{1.34702, 6.29049}	{2.13116, 13.6579}	{{2.08003, 13.1443}, {2.2143, 14.4309}}	{58.2132, 117.119}	inf	82.	343.46	{130., 534.}	319.009	0.534999	newly	86.	157.841	{53., 235.}	98.1912	0.251611	Reff	0.773998	0.41898	{0.366501, 0.450951}	46.0745	ar	4560.	8274.78	{8103., 8493.}	81.4644
30	R0	{1.37931, 6.78741}	{2.11728, 14.2489}	{{2.12155, 14.3165}, {2.12155, 14.3165}}	{53.5027, 109.932}	inf	58.	297.426	{103., 435.}	412.804	0.591371	newly	51.	126.721	{41., 184.}	156.509	0.314743	Reff	0.764552	0.4133	{0.369786, 0.440683}	45.9421	ar	4667.	8266.26	{8145., 8381.}	77.1215
31	R0	{1.16062, 4.47529}	{1.69756, 6.15907}	{{1.69756, 6.15907}, {1.69756, 6.15907}}	{46.2632, 37.6239}	inf	38.	66.125	{14., 150.}	113.882	0.323835	newly	49.	59.85	{10., 140.}	88.2653	0.371358	Reff	0.82288	0.533636	{0.501968, 0.558158}	35.1501	ar	3037.	6932.93	{6787., 7053.}	128.282
32	R0	{1.37647, 18.7122}	{1.6411, 19.8325}	{{1.6411, 19.8325}, {1.6411, 19.8325}}	{19.2253, 5.987}	inf	359.	376.027	{122., 752.}	49.5822	0.225802	newly	137.	123.514	{41., 241.}	48.0371	0.25426	Reff	0.886034	0.661119	{0.563882, 0.804139}	26.1122	ar	4824.	6654.59	{6451., 6871.}	37.9476
33	R0	{1.35084, 6.08841}	{2.2277, 15.0861}	{{2.2277, 15.0861}, {2.2277, 15.0861}}	{64.9122, 147.784}	inf	48.	531.228	{201., 922.}	1006.73	0.975456	newly	49.	220.596	{85., 400.}	350.197	0.577162	Reff	0.722834	0.432588	{0.344625, 0.480292}	45.1363	ar	4759.	8494.67	{8408., 8602.}	78.4969
34	R0	{1.36912, 6.316}	{2.04544, 14.1646}	{{2.04423, 14.1631}, {2.04423, 14.1631}}	{49.3978, 124.266}	inf	145.	475.143	{219., 767.}	228.191	0.470623	newly	140.	208.755	{93., 336.}	63.484	0.202288	Reff	0.820103	0.461982	{0.404962, 0.526185}	43.6678	ar	4537.	8105.24	{7975., 8197.}	78.6477
35	R0	{1.35831, 7.09038}	{2.2119, 14.1654}	{{2.20345, 14.058}, {2.21411, 14.2212}}	{62.8419, 99.7827}	inf	73.	259.274	{87., 465.}	256.407	0.458235	newly	59.	108.484	{38., 204.}	101.148	0.26097	Reff	0.752504	0.374127	{0.34208, 0.412267}	50.2823	ar	4667.	8459.39	{8372., 8543.}	81.2596
36	R0	{1.28315, 5.89938}	{2.25358, 12.7711}	{{2.18735, 12.1087}, {2.31231, 13.3585}}	{75.6286, 116.482}	inf	40.	246.906	{97., 370.}	517.266	0.681143	newly	43.	112.094	{38., 166.}	165.407	0.320038	Reff	0.715356	0.358451	{0.317018, 0.38738}	49.892	ar	4460.	8552.94	{8398., 8709.}	91.7699
37	R0	{1.19303, 2.75113}	{1.69756, 6.15907}	{{1.69756, 6.15907}, {1.69756, 6.15907}}	{42.2898, 123.874}	inf	5.	39.325	{7., 84.}	691.5	0.746955	newly	9.	33.15	{7., 72.}	279.444	0.472617	Reff	0.839297	0.528026	{0.499422, 0.554253}	37.0871	ar	3142.	6932.93	{6787., 7053.}	120.653
38	R0	{1.36033, 7.8088}	{2.15159, 13.48}	{{2.15363, 13.5067}, {2.15363, 13.5067}}	{58.1671, 72.6252}	inf	165.	268.804	{111., 603.}	78.6928	0.217807	newly	127.	122.353	{46., 261.}	51.212	0.23709	Reff	0.778789	0.392641	{0.358795, 0.440848}	49.5832	ar	4941.	8343.18	{8269., 8430.}	68.856
39	R0	{1.27353, 3.70845}	{1.95431, 9.79172}	{{1.93539, 9.63533}, {1.95791, 9.82151}}	{53.4559, 164.038}	inf	7.	99.4483	{35., 202.}	1320.69	1.04777	newly	14.	57.7241	{20., 121.}	316.236	0.519996	Reff	0.800796	0.432993	{0.412432, 0.464612}	45.9296	ar	3725.	7856.03	{7744., 7949.}	110.9
40	R0	{1.19983, 12.1486}	{1.59433, 19.1171}	{{1.59433, 19.1171}, {1.59433, 19.1171}}	{32.8797, 57.3605}	inf	140.	307.367	{111., 628.}	127.405	0.305895	newly	71.	106.867	{35., 233.}	79.3427	0.25174	Reff	0.902632	0.681454	{0.581133, 0.731797}	27.633	ar	3619.	6407.07	{6165., 6603.}	77.0397
41	R0	{1.20935, 2.67169}	{1.80312, 5.98216}	{{1.80312, 5.98216}, {1.80312, 5.98216}}	{49.0983, 123.909}	inf	0.001	15.5556	{4., 35.}	1.55546×10^{6}	2.01767	newly	0.1	13.4444	{3., 33.}	13348.9	1.93516	Reff	0.794664	0.480207	{0.4506, 0.505415}	39.5711	ar	3429.	7350.33	{7225., 7517.}	114.358
42	R0	{1.23313, 4.74781}	{1.86902, 12.6353}	{{1.86902, 12.6353}, {1.86902, 12.6353}}	{51.5672, 166.129}	inf	65.	249.2	{97., 368.}	283.863	0.496139	newly	90.	119.933	{40., 184.}	65.358	0.220107	Reff	0.791546	0.492537	{0.441463, 0.546098}	37.7754	ar	3835.	7602.69	{7482., 7706.}	98.2448
43	R0	{1.26105, 2.39713}	{1.93419, 8.56054}	{{1.93419, 8.56054}, {1.93419, 8.56054}}	{53.3793, 257.116}	inf	1.	69.6585	{25., 109.}	6865.85	1.73669	newly	1.	46.122	{18., 76.}	4512.2	1.55612	Reff	0.791561	0.437669	{0.406373, 0.464592}	44.7081	ar	3723.	7790.85	{7670., 7956.}	109.263
44	R0	{1.36948, 8.88006}	{2.03201, 13.7734}	{{2.01902, 13.4708}, {2.04423, 14.1631}}	{48.3785, 55.1044}	inf	166.	532.033	{219., 832.}	222.161	0.446081	newly	127.	243.492	{103., 367.}	101.949	0.265221	Reff	0.844011	0.490545	{0.427234, 0.534435}	42.3917	ar	4437.	8063.72	{7975., 8165.}	81.7381
45	R0	{1.34882, 3.3791}	{2.12981, 8.24814}	{{2.12684, 8.23415}, {2.12684, 8.23415}}	{57.9014, 144.093}	inf	6.	103.255	{30., 213.}	1620.91	1.12197	newly	9.	67.3636	{20., 138.}	648.485	0.756513	Reff	0.755069	0.37561	{0.350929, 0.403887}	50.2548	ar	4422.	8300.55	{8172., 8410.}	87.7102
46	R0	{1.23774, 4.34917}	{1.93462, 9.38215}	{{1.93462, 9.38215}, {1.93462, 9.38215}}	{56.3026, 115.723}	inf	30.	167.2	{58., 276.}	457.333	0.678195	newly	38.	104.225	{40., 169.}	175.592	0.374499	Reff	0.787698	0.456561	{0.409366, 0.49062}	42.0386	ar	3685.	7776.53	{7616., 7920.}	111.032
47	R0	{1.34327, 4.78811}	{2.1961, 12.1962}	{{2.18735, 12.1087}, {2.18735, 12.1087}}	{63.4889, 154.718}	inf	76.	419.455	{167., 733.}	451.914	0.671807	newly	94.	205.582	{79., 341.}	125.513	0.300498	Reff	0.773992	0.40263	{0.353694, 0.473561}	47.9801	ar	4517.	8430.6	{8356., 8523.}	86.6416
48	R0	{1.24366, 4.25814}	{1.91895, 11.3485}	{{1.88025, 10.9611}, {1.98732, 12.0157}}	{54.2984, 166.514}	inf	38.	329.333	{146., 514.}	766.667	0.853237	newly	50.	176.314	{75., 274.}	254.196	0.463548	Reff	0.803653	0.496188	{0.433434, 0.555732}	39.1556	ar	3678.	7742.43	{7543., 7994.}	110.507
49	R0	{1.36017, 7.5415}	{1.68954, 11.6019}	{{1.68954, 11.6019}, {1.68954, 11.6019}}	{24.2154, 53.8407}	inf	152.	285.923	{110., 488.}	102.716	0.264253	newly	140.	159.205	{61., 301.}	61.337	0.249681	Reff	0.945454	0.629215	{0.531529, 0.708593}	37.423	ar	4178.	6867.97	{6664., 7049.}	64.3843
50	R0	{1.25333, 6.63578}	{1.76123, 10.8087}	{{1.76659, 10.896}, {1.76659, 10.896}}	{40.5243, 62.8849}	inf	49.	110.925	{41., 198.}	135.357	0.275779	newly	43.	63.125	{23., 106.}	79.593	0.229479	Reff	0.846499	0.516775	{0.476096, 0.560009}	38.9515	ar	3401.	7189.35	{6948., 7340.}	111.389
